# Characteristics of a multigene assay (MammaPrint/Blueprint) to predict early recurrence of hormone receptor-positive, HER2-negative breast cancer: a case‒control study (WJOG16722B)

**DOI:** 10.1007/s12282-026-01847-2

**Published:** 2026-03-26

**Authors:** Rurina Watanuki, Hitomi Sakai, Yuri Kimura, Atsushi Yoshida, Yukinori Ozaki, Akemi Kataoka, Natsue Uehiro, Atsushi Fushimi, Hidenori Kamio, Takashi Ikeno, Masashi Wakabayashi, Mayumi Iida, Tsutomu Kawaguchi, Junji Tsurutani, Toshimi Takano

**Affiliations:** 1 Department of Breast Surgery, National Cancer Center Hospital East, 6-5-1 Kashiwanoha, Kashiwa-Shi, Chiba 277-8577 Japan; 2 Advanced Cancer Translational Research Institute, Showa Medical University, 1-5-8 Hatanodai, Shinagawa-Ku, Tokyo 142-8555 Japan; 3 Department of Breast Surgical Oncology, St. Luke s International Hospital, 9-1 Akashicho, Chuo-Ku, Tokyo 104-8560 Japan; 4 Breast Oncology Center, Cancer Institute Hospital of the Japanese Foundation for Cancer Research, 3-8-31 Ariake, Koto-Ku, Tokyo 135-8550 Japan; 5 Department of Surgery, The Jikei University School of Medicine, 3-19-18 Nishi-Shinbashi, Minato-Ku, Tokyo 105-8471 Japan; 6 Department of Breast Surgery, Tokyo Metropolitan Cancer and Infectious Diseases Center Komagome Hospital, 3-18-22 Honkomagome, Bunkyo-Ku, Tokyo 113-8677 Japan; 7 Clinical Research Support Office, National Cancer Center Hospital East, 6-5-1 Kashiwanoha, Kashiwa-Shi, Chiba 277-8577 Japan; 8 Biostatistics Division, Center for Research Administration and Support, National Cancer Center, 6-5-1 Kashiwanoha, Kashiwa-Shi, Chiba 277-8577 Japan; 9 Japan Drug Development and Medical Affairs, Eli Lilly Japan, 5-1-28 Isogamidori, Chuo-Ku, Kobe-Shi, Hyogo 651-0086 Japan

**Keywords:** HR-positive HER2-negative breast cancer, Early recurrence, MammaPrint, BluePrint, Genomic risk

## Abstract

**Background:**

Hormone receptor-positive, HER2-negative (HR + /HER2-) breast cancer generally has a favorable prognosis; however, early postoperative recurrence markedly reduces survival. Accurate prediction of early recurrence is crucial for personalizing treatment. This case–control study compared MammaPrint (MP) and BluePrint (BP) results between early recurrence patients and matched controls.

**Methods:**

Patients were selected from our previous study, the WJOG15721B cohort (n = 2732). Those with recurrence within three years after surgery were randomly extracted, and controls matched for institution, clinical stage, and number of pathological lymph node metastases were included (n = 124). Tumor samples underwent MP and BP assays to classify recurrence risk and molecular subtypes.

**Results:**

Of 115 submitted tumor samples, 85 were analyzed successfully (43 early recurrence, 42 no recurrence). High-risk MP classification was significantly more frequent in early recurrence patients (79.1% vs. 40.5%, p < 0.001), and Luminal B BP subtype was more common in early recurrence patients (79.1% vs. 38.1%, p < 0.001). High MP risk was associated with high Ki-67 levels and higher nuclear grade. Integrating clinical and genomic risk enhanced prognostic precision: patients with both clinical and genomic high risk had the highest recurrence rate (100%), those with low clinical and genomic risk had the lowest (28.1%), and patients with low clinical but high genomic risk showed an intermediate recurrence rate (57.5%).

**Conclusions:**

Compared with patients without recurrence, those with early recurrence showed a significantly higher prevalence of high-risk MP results and Luminal B BP subtype. High-risk MP/Luminal B BP subtype suggested an association with early recurrence in patients with HR + /HER2- early breast cancer.

**Supplementary Information:**

The online version contains supplementary material available at 10.1007/s12282-026-01847-2.

## Introduction

Hormone receptor-positive, human epidermal growth factor receptor 2-negative (HR +/HER2-) breast cancer has a good prognosis, but patients with early recurrence are difficult to treat and have a relatively poorer prognosis than those of with late recurrence. The 5-year survival of patients who experience recurrence within 3 years after initial treatment has been reported to be 29% [1]. As new treatment options for the postoperative treatment of HR +/HER2- breast cancer other than chemotherapy, such as cyclin-dependent kinase 4 and 6 (CDK4/6) inhibitors, are emerging to reduce the risk of recurrence, it is important to estimate the risk of recurrence in detail to personalize treatment. We conducted a retrospective study to identify risk factors for early recurrence in patients with HR +/HER2- early breast cancer who received adjuvant endocrine therapy by evaluating the correlations among clinicopathological characteristics, perioperative treatment, and prognosis (WJOG15721B/RealisE, UMIN000047049). The results revealed that younger age, nuclear grade, vascular invasion, tumor size, and number of lymph node metastases were independent risk factors for early recurrence [2].

While clinicopathological factors are essential for predicting postoperative recurrence, a multigene assay for predicting recurrence has been clinically applied in HR +/HER2- breast cancer patients. MammaPrint, a multigene assay, classifies patients into high- and low-risk groups on the basis of the expression patterns of 70 genes and predicts recurrence. The MINDACT trial demonstrated that patients with high clinical risk and low genomic risk according to MammaPrint had excellent distant metastasis-free survival after endocrine therapy alone. As a result, adjuvant chemotherapy can be omitted in 46% of patients with high-risk breast cancer without affecting survival outcomes [3, 4]. Along with MammaPrint, BluePrint is a test to identify biological differences unique to individual breast cancers by capturing activated downstream molecular pathways on the basis of the expression of 80 genes and classifying them into four subtypes (Luminal A-type, Luminal B-type, HER2-type, and Basal-type) using formalin-fixed, paraffin-embedded specimens of the same primary tumor [5, 6].

Although MammaPrint can predict the risk of postoperative recurrence of early breast cancer and identify patients for whom adjuvant chemotherapy could be safely omitted, there have been no reports evaluating the results of MammaPrint and/or BluePrint in patients with HR +/HER2- breast cancer that recurred within 3 years after surgery. This study aimed to compare MammaPrint and BluePrint results between early recurrence cases and nonrecurrence controls from the WJOG15721B study.

## Patients and methods

### Patients

Patients aged ≥ 20 years with histologically confirmed HR +/HER2- clinical stage II-III invasive breast cancer who received adjuvant endocrine therapy between 2012 and 2017 (n = 2,732) were included in a retrospective cohort study (WJOG15721B study) across five institutions in Japan. The study methods and results were published previously [2]. Among the enrolled patients, we recruited patients for the WJOG16722B/RealisE-TR study (UMIN000050930) using the following method.

Approximately 70 patients who experienced recurrence within 3 years after surgery (early recurrence cases) were randomly selected from the patients enrolled in WJOG15721B study, within a retrospective cohort in which recurrence outcomes had already been determined. We defined early recurrence as recurrence occurring within 3 years after surgery because previous studies have used various definitions (e.g., 2.5 years from diagnosis or 5 years after surgery) without a clear consensus [7, 8], and considering that endocrine relapse within 2 years is regarded as primary endocrine resistance in international guidelines [9] and that the benefit of adjuvant abemaciclib persists beyond the 2-year treatment period [10]. Afterward, data from approximately 70 patients who did not experience recurrence (control) were identified 1:1 matching based on three factors: institution, clinical stage, and the number of pathologically positive lymph nodes. If matching was not possible, the case was excluded from the analysis. Importantly, the matching was performed by a biostatistician prior to sample testing and without knowledge of the outcome status. Furthermore, since the parent study (WJOG15721B) included approximately 2,700 cases, no missing data occurred due to an inability to identify matched controls for the selected cases.

Informed consent was obtained, but for cases where the patient had passed away or relocated with an unknown contact address, an opt-out procedure was conducted.

### Molecular signature tests

Molecular signature testing was conducted using needle biopsy samples obtained at the time of diagnosis or tissue samples obtained at the time of surgery. MammaPrint® and BluePrint® (Agendia, Inc.) molecular signature tests were performed in a validated laboratory. For the MammaPrint® assay, each case was assigned to a recurrence risk category, along with a molecular subgroup determined by the BluePrint® assay." Tumors were classified with MammaPrint as low risk [0.000 < MammaPrint index (MPI) ≤ 1.000] or high risk (−1.000 ≤ MPI < 0.000). Among the low-risk patients, those with scores in the range of + 0.356 to + 1.000 were defined as being at ultralow risk. BluePrint categorizes tumors by subtype (Luminal, HER2, and Basal). Together, MammaPrint and BluePrint further stratified luminal-type tumors as Luminal A-type (MammaPrint low risk) or Luminal B-type (MammaPrint high risk) tumors.

### Endpoints

The primary endpoint was the proportion of high-risk patients according to MammaPrint. The secondary endpoints were the proportions of BluePrint Basal-type and other molecular subtypes identified by BluePrint and the relationships between MammaPrint/BluePrint and clinicopathological factors.

### Categorization into risk groups

Patients were classified into low or high clinical risk groups in an exploratory manner on the basis of the total nomogram score we developed in the WJOG1572B study to predict the risk of early recurrence [2]. The cutoff value was set at 250 points, which was based on the score corresponding to a 3-year invasive disease-free survival of 83.4% in the endocrine therapy-alone group in the monarchE trial [10, 11]. Additionally, MammaPrint high-risk and low-risk patients were categorized into high genomic high-risk and low genomic risk groups.

### Statistical analysis

The MPI obtained via MammaPrint for the early recurrence and no recurrence groups was summarized and compared between groups using the Wilcoxon rank sum test. For each group, we estimated the proportion of MammaPrint high risk cases and the 95% confidence interval on the basis of the binomial distribution. To evaluate the association between high-risk MammaPrint results and recurrence, Fisher’s exact test was performed. Missing values were not imputed and were excluded from the analysis. BluePrint subtypes (Luminal A, Luminal B, HER2, and Basal types) were summarized for the early recurrence and no recurrence groups using Fisher's direct probability test. The associations between MammaPrint index values and clinicopathological factors were evaluated using t tests and analysis of variance (ANOVA), and the associations between the MammaPrint high-risk/low-risk results and clinicopathological factors were evaluated on the basis of odds ratios (ORs) and 95% confidence intervals (CIs) by logistic regression analysis. The associations between BluePrint subtype and clinicopathological factors were analyzed similarly. In this study, pair-matched analysis could not be performed due to the unavailability of some samples or the failure of genomic analysis after submission. All analyses were conducted with SAS (version 9.4; Cary, NC, USA).

## Results

### Patient clinical characteristics

**Fig. 1:**
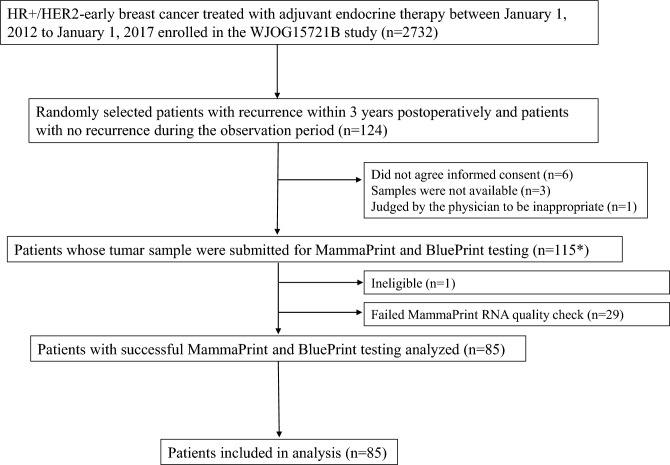
Flow chart of patient selection. ^*^Of the 124 eligible candidates, 10 were excluded, but one ineligible patient's specimen was submitted, for a total of 115 samples. *HR* hormone receptor, *HER2* human epidermal growth factor 2

A flow chart of patient selection is presented in Fig. 1. Among the 2732 patients included in the WJOG15721B study, 62 patients with early recurrence and 62 matched patients without recurrence were selected as candidates. MammaPrint/BluePrint testing was performed on 115 patients. Twenty-nine patients failed to pass the MammaPrint RNA quality check, 86 (74.8%) were successfully analyzed, and 85 were ultimately included in the study analysis, except one who was ineligible.

The patient background and clinicopathological characteristics of the study population are shown in Table 1 and Supplementary Table 1. The median age was 53.5 years in the no recurrence group and 47 years in the early recurrence group. Twenty-two patients (52.4%) in the no recurrence group and 26 (60.5%) in the early recurrence group were premenopausal. In the nonrecurrence group, 32 (76.2%) patients had clinical stage IIA disease, 4 (9.5%) patients had stage IIB disease, and 6 (14.3%) patients had stage III disease; in the early recurrence group, 31 (72.1%), 7 (16.3%), and 5 (11.6%) patients had clinical stage IIA, IIB and III disease, respectively. The number of pathological lymph node metastases was as follows: 16 (38.1%) had none, 19 (45.2%) had 1–3, and 7 (16.7%) had ≥ 4 in the no recurrence group and 15 (34.9%) had none, 19 (44.2%) had 1–3, and 9 (20.9%) had ≥ 4 in the early recurrence group. There was a trend toward a high proportion of nuclear grade 2 (n = 25, 58.1%) and grade 3 (n = 11, 25.6%) patients in the early recurrence group compared with grade 2 (n = 12, 28.6%) and grade 3 (n = 8, 19.0%) patients in the no recurrence group. There was a greater tendency for patients in the early recurrence group (23.3%) to receive neoadjuvant chemotherapy (no recurrence group: 9.5%), but the proportion of patients who received adjuvant chemotherapy did not differ between the no recurrence (39.5%) and early recurrence groups (40.5%) (Table 2).

**Table 1:** Patient characteristics and clinicopathological features of the study population

	No recurrence (n = 42)	Early recurrence (n = 43)	Total (n = 85)
*Age, years*			
Median (range)	53.5 (32–78)	47 (33–86)	48 (32–86)
Performance status^*1^	(n = 27)	(n = 23)	
0	27 (100)	23 (100)	50
1	0 (0)	0 (0)	0
*Menopausal status*			
Premenopausal	22 (52.4)	26 (60.5)	48
Postmenopausal	20 (47.6)	16 (37.2)	36
Unknown	0 (0)	1 (2.3)	1
*Clinical stage*			
IIA	32 (76.2)	31 (72.1)	63
IIB	4 (9.5)	7 (16.3)	11
IIIA	3 (7.1)	3 (7.0)	6
IIIB	3 (7.1)	2 (4.7)	5
IIIC	0 (0)	0 (0)	0
*Nuclear grade*			
1	22 (52.4)	4 (9.3)	26
2	12 (28.6)	25 (58.1)	37
3	8 (19.0)	11 (35.6)	19
Unknown	0 (0)	3 (7.0)	3
*Pathological tumor size (cm)*			
< 2	11 (26.2)	5 (11.6)	16
2 to less than 5	28 (66.7)	29 (67.4)	57
≥ 5	3 (7.1)	7 (16.3)	10
Unknown	0 (0)	2 (4.7)	2
*Number of pathological lymph node metastases*			
0	16 (38.1)	15 (34.9)	31
1–3	19 (45.2)	19 (44.2)	38
4–9	4 (9.5)	5 (11.6)	9
≥ 10	3 (7.1)	4 (9.3)	7

^*^1 Data represent aggregated results from 50 cases obtained from two institutions

**Table 2:** Perioperative treatment details for the study population

	No recurrence (n = 42)	Early recurrence (n = 43)	Total (n = 85)
*Neoadjuvant chemotherapy*			
No	38 (90.5)	33 (76.7)	71
Yes	4 (9.5)	10 (23.3)	14
Anthracyclines	4 (9.5)	10 (23.3)	14
Taxanes	4 (9.5)	10 (23.3)	14
*Adjuvant chemotherapy*			
No	25 (59.5)	26 (60.5)	51
Yes	17 (40.5)	17 (39.5)	34
Anthracyclines	15 (35.7)	16 (37.3)	31
Taxanes	17 (40.5)	17 (39.5)	34
Others	2 (4.8)	2 (4.7)	4
*Adjuvant endocrine therapy*			
No	0 (0)	0 (0)	0
Yes	42 (100)	43 (100)	85

Patient characteristics and clinicopathological findings comparing the successful analysis group (n = 85) and the unsuccessful analysis group (n = 29) are presented in Supplementary Table 3.

### Characteristics of MammaPrint and BluePrint

**Fig. 2:**
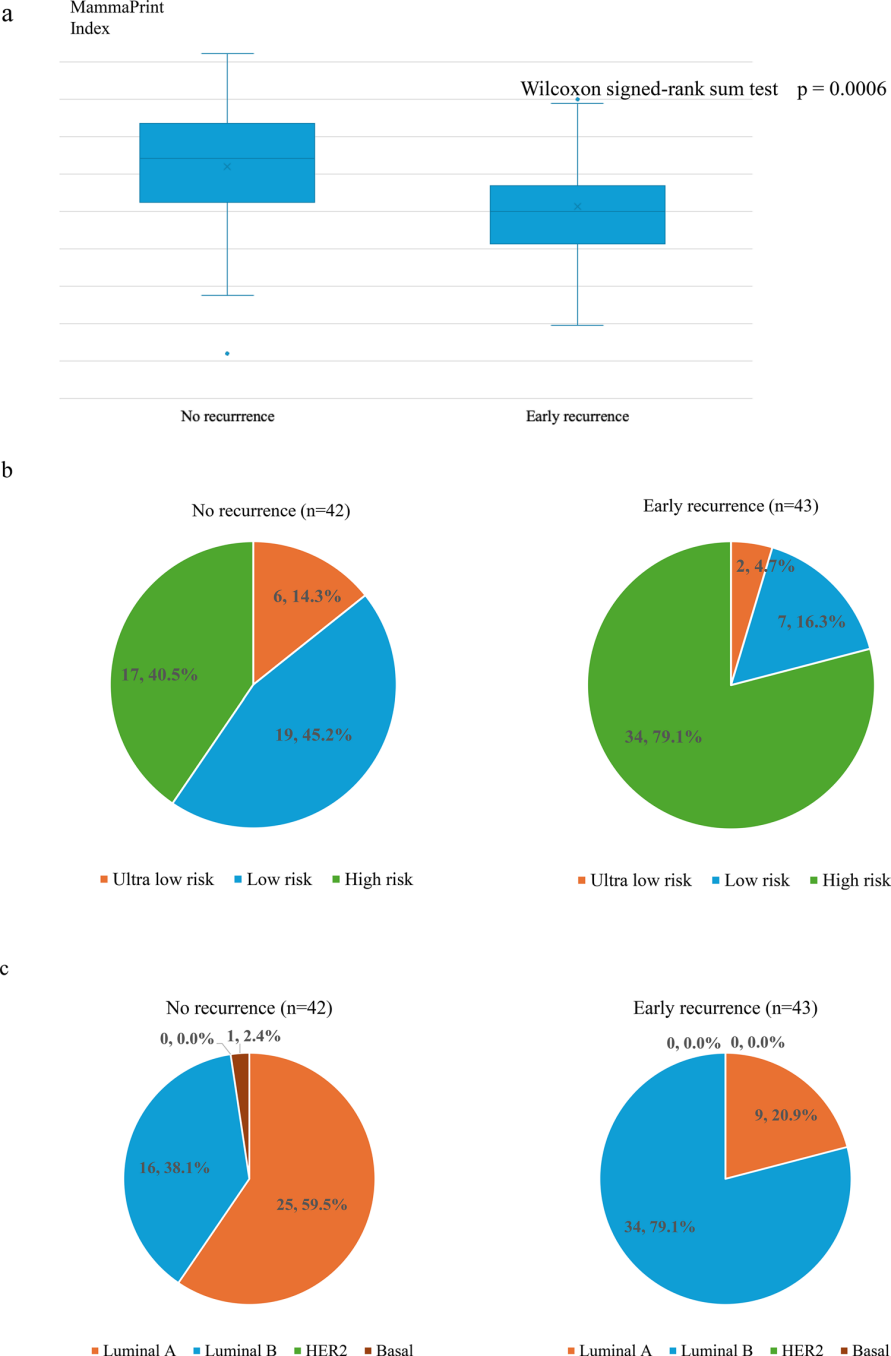
**a** Distribution of the MammaPrint index results in the early recurrence and no recurrence groups. **b** MammaPrint risk classification for the early recurrence and no recurrence groups. **c** Molecular subtyping based on the BluePrint index in the early recurrence and no recurrence groups

The MPI value was greater in the no recurrence group than in the early recurrence group, with a value of 0.086 (median, IQR: −0.1420.269; range: −0.9600.645) in the no recurrence group and −0.261 (median, IQR: −0.372–0.063; range: −0.8100.402) in the early recurrence group (p < 0.001) (Fig. 2a). In the nonrecurrence group, 17 patients (40.5%) were classified as high risk, 19 patients (45.2%) as low risk, and 6 patients (14.3%) as ultralow risk (Fig. 2b). In contrast, in the early recurrence group, 34 patients (79.1%) were classified as high risk, 7 patients (16.3%) as low risk, and 2 patients (4.7%) as ultralow risk (Fig. 2b). The proportion of patients at high risk on the basis of MammaPrint was 79.1% [95% confidence interval (CI) 64.0–90.0] in the early recurrence group and 40.5% (95% CI 25.6–56.7) in the no recurrence group, with a statistically significant difference (p < 0.001). Only one patient in the nonrecurrence group had the basal type (Fig. 2c). The percentage of Luminal B-type tumors was 79.1% (95% CI 64.0–90.0) in the early recurrence group and 38.1% (95% CI 23.6–54.4) in the no recurrence group, with a significant difference (p < 0.001) (Fig. 2c).

We performed the planned analysis to examine whether any subgroup exhibited high genomic risk, focusing on covariates that have been identified as prognostic or predictive factors for chemotherapy in previous studies (Table 3). There were significant associations of high-risk MammaPrint results with Ki-67 levels [≥ 30% vs. < 14%: odds ratio (OR) 26.43, 95% CI 3.03–230.16, p = 0.003] and nuclear grade (Grade 2 vs. Grade 1: OR 5.30, 95% CI 1.77–15.91, p = 0.003; Grade 3 vs. Grade 1: OR 12.26, 95% CI 2.84–52.87, p < 0.001). There were also significant interactions of Luminal B-type results in the BluePrint assay with Ki-67 levels (≥ 30% vs. < 14%: odds ratio (OR) 15.00, 95% CI 2.24–100.51, p = 0.005) and nuclear grade (Grade 2 vs. Grade 1: OR 5.30, 95% CI 1.77–15.91, p = 0.003; Grade 3 vs. Grade 1: OR 8.96, 95% CI 2.26–35.44, p = 0.002) (Table 4).

**Table 3:** Associations between high-risk MammaPrint classification and clinicopathological factors

Factor	Level	n	Proportion of MammaPrint high risk	OR (95% CI)^*1^	P value^*2^
Age	20–39 years	11	9 (81.8%)	1	(0.387)
	40–69 years	62	35 (56.5%)	0.34 (0.07–1.58)	0.169
	≥ 70 years	12	7 (58.3%)	0.36 (0.06–2.29)	0.278
Menopausal status	Premenopausal	48	29 (60.4%)	1	0.847
	Postmenopausal	36	21 (58.3%)	0.92 (0.38–2.21)	
Bilateral breast cancer	No	84	50 (59.5%)	1	0.746
	Yes	1	1 (100%)	2.14 (0.02–211.12)	
ER	< 1%	-	-	-	-
	1–9%	-	-	-	-
	≥ 10%	46	26 (56.5%)	1	0.491
	Unknown	39	25 (64.1%)	1.36 (0.57–3.26)	
PgR	< 1%	5	2 (40.0%)	1	(0.660)
	≥ 1%	24	14 (58.3%)	1.93 (0.27–13.65)	0.508
	Unknown	56	35 (62.5%)	2.31 (0.36–14.83)	0.377
HER2	0	40	22 (55.0%)	1	(0.522)
	1 +	34	23 (67.7%)	1.68 (0.65–4.34)	0.284
	2 +	11	6 (54.6%)	0.97 (0.25–3.71)	0.966
Ki-67	< 14%	10	3 (30.0%)	1	(0.015)
	14–29%	9	3 (33.3%)	1.15 (0.17–7.79)	0.883
	≥ 30%	19	18 (94.7%)	26.43 (3.03–230.16)	0.003
	Unknown	47	27 (57.5%)	2.87 (0.67–12.26)	0.154
Nuclear grade	Grade 1	26	7 (26.9%)	1	(0.002)
	Grade 2	37	25 (67.6%)	5.30 (1.77–15.91)	0.003
	Grade 3	19	16 (84.2%)	12.26 (2.84–52.87)	0.001
	Unknown	3	3 (100%)	18.21 (0.54–620.16)	0.107
Histological grade	Grade1	5	3 (60.0%)	1	(0.514)
	Grade2	6	2 (33.3%)	0.40 (0.04–4.55)	0.458
	Grade3	2	2 (100%)	3.57 (0.06–220.19)	0.545
Lymphatic invasion	No	34	17 (50.0%)	1	0.134
	Yes	51	34 (66.7%)	1.97 (0.81–4.79)	
Vascular invasion	No	73	45 (61.6%)	1	0.644
	Yes	11	6 (54.6%)	0.74 (0.21–2.65)	
Pathological tumor size	< 2 cm	16	8 (50.0%)	1	(0.646)
	2 cm to less than 5 cm	57	34 (59.7%)	1.47 (0.48–4.47)	0.499
	≥ 5 cm	10	7 (70.0%)	2.14 (0.41–11.19)	0.366
Number of pathological lymph node metastases	0	31	18 (58.1%)	1	(0.450)
	1–3	38	21 (55.3%)	0.90 (0.34–2.34)	0.823
	≥ 4	16	12 (75.0%)	2.03 (0.54–7.59)	0.294
NAC	No	71	39 (54.9%)	1	0.061
	Yes	14	12 (85.7%)	4.11 (0.94–18.09)	

*OR* odds ratio, *CI* confidence interval, *NAC* neoadjuvant chemotherapy

^*^1 Odds ratios estimated via a logistic regression model using Firth's penalized maximum likelihood

^*^2 Wald test p values for odds ratios estimated via a logistic regression model

**Table 4:** Association between Luminal B-type disease identified by BluePrint and clinicopathological factors

Factor	Level	n	Proportion of BluePrint Luminal B-type	OR (95% CI)^*1^	P value^*2^
Age	20–39 years	11	9 (81.8%)	1	(0.346)
	40–69 years	62	34 (54.8%)	0.32 (0.07–1.48)	0.145
	≥ 70 years	12	7 (58.3%)	0.36 (0.06–2.29)	0.278
Menopausal status	Premenopausal	48	28 (58.3%)	1	0.996
	Postmenopausal	36	21 (58.3%)	0.10 (0.42–2.40)	
Bilateral breast cancer	No	84	49 (58.3%)	1	0.732
	Yes	1	1 (100%)	2.23 (0.02–218.41)	
ER	< 1%	-	-	-	-
	1–9%	-	-	-	-
	≥ 10%	46	26 (56.5%)	1	0.650
	Unknown	39	24 (61.5%)	1.22 (0.51–2.91)	
PgR	< 1%	5	2 (40.0%)	1	(0.720)
	≥ 1%	24	14 (58.3%)	1.93 (0.27–13.65)	0.508
	Unknown	56	34 (60.7%)	2.15 (0.34–13.75)	0.420
HER2	0	40	22 (55.0%)	1	(0.681)
	1 +	34	22 (64.7%)	1.48 (0.58–3.78)	0.413
	2 +	11	6 (54.6%)	0.97 (0.26–3.71)	0.966
Ki-67	< 14%	10	3 (30.0%)	1	(0.021)
	14–29%	9	3 (33.3%)	1.15 (0.17–7.79)	0.883
	≥ 30%	19	17 (89.5%)	15.00 (2.24–100.51)	0.005
	Unknown	47	27 (57.5%)	2.87 (0.67–12.26)	0.154
Nuclear grade	Grade 1	26	7 (26.9%)	1	(0.004)
	Grade 2	37	25 (67.6%)	5.30 (1.77–15.91)	0.003
	Grade 3	19	15 (79.0%)	8.96 (2.26–35.44)	0.002
	Unknown	3	3 (100%)	18.21 (0.54–620.04)	0.107
Histological grade	Grade1	5	3 (60.0%)	1	(0.514)
	Grade2	6	2 (33.3%)	0.40 (0.04–4.55)	0.458
	Grade3	2	2 (100%)	3.57 (0.06–220.19)	0.545
Lymphatic invasion	No	34	17 (50.0%)	1	0.188
	Yes	51	33 (64.7%)	1.81 (0.75–4.38)	
Vascular invasion	No	73	44 (60.3%)	1	0.707
	Yes	11	6 (54.6%)	0.78 (0.22–2.80)	
Pathological tumor size	< 2 cm	16	7 (43.8%)	1	(0.428)
	2 cm to less than 5 cm	57	34 (59.7%)	1.86 (0.61–5.70)	0.277
	≥ 5 cm	10	7 (70.0%)	2.72 (0.52–14.23)	0.237
Number of pathological lymph node metastases	0	31	17 (54.8%)	1	(0.409)
	1–3	38	21 (55.3%)	1.02 (0.39–2.64)	0.971
	≥ 4	16	12 (75.0%)	2.30 (0.62–8.59)	0.215
NAC	No	71	39 (54.9%)	1	0.141
	Yes	14	11 (78.6%)	2.70 (0.72–10.16)	

*OR* odds ratio, *CI* confidence interval, *NAC* neoadjuvant chemotherapy

^*^1 Odds ratios estimated via a logistic regression model using Firth's penalized maximum likelihood

^*^2 Wald test p values for odds ratios estimated via a logistic regression model

### Comparison of genomic risk and clinical risk

**Fig. 3:**
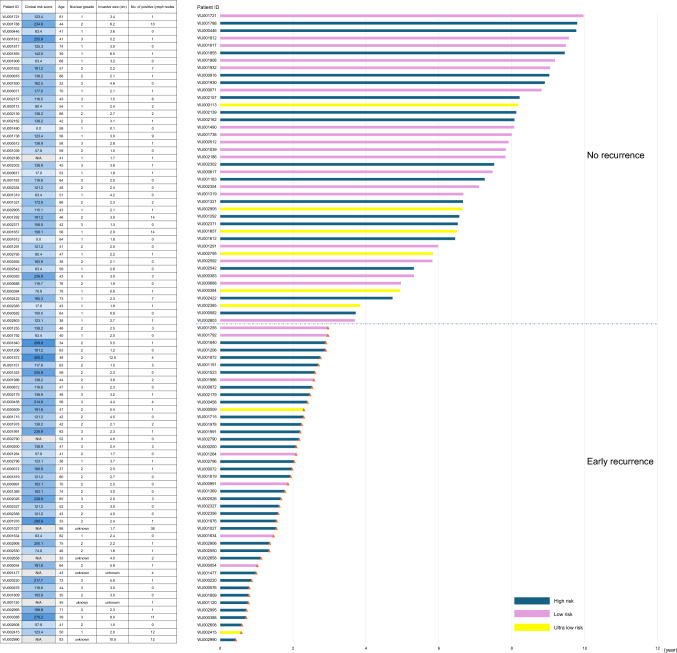
Swimmer plot of MammaPrint genomic risk in early and no recurrence groups (n = 85). In addition to the swimmer plot, the clinical scores according to the nomogram, age, nuclear grade, pathological invasive tumor size, and number of pathological lymph node metastases are shown in the table

Among the 85 patients analyzed in this study, 7 patients were excluded because they were not subjected to multivariable analysis in the WJOG15721B study due to missing values and thus could not have their total points in the nomogram appropriately calculated. The remaining 78 patients were categorized into four groups: clinical high-risk/genomic high-risk, clinical high-risk/genomic low-risk, clinical low-risk/genomic high-risk, and clinical low-risk/genomic low-risk (Table 5). Among patients with both high clinical and genomic risk, the rate of early recurrence was 100% (5/5). In contrast, those with both low clinical and genomic risk had a low risk of early recurrence, at 28.1% (9/32). Only one patient had high clinical risk and low genomic risk, and this patient did not experience early recurrence. Notably, among patients with low clinical risk but high genomic risk, 57.5% (23/40) experienced early recurrence, which is numerically higher than the 28.1% (9/32) recurrence rate observed in those with both low clinical risk and low genomic risk. Furthermore, the 85 patients were divided into an early recurrence group and a no recurrence group, and the breakdown of genomic risk was illustrated using a swimmer plot (Fig. 3). Remarkably, of the 43 patients with early recurrence, 15 (34.9%) were negative for lymph node metastasis, and 13 (30.2%) of them were classified as having low clinical risk.

**Table 5:** Clinical risk and genomic risk in the early recurrence and no recurrence groups

	Clinical high risk/Genomic high risk n = 5 (%)	Clinical high risk/Genomic low risk n = 1 (%)	Clinical low risk/Genomic high risk n = 40 (%)	Clinical low risk/Genomic low risk n = 32 (%)
Early recurrence	5 (100)	0 (0)	23 (57.5)	9 (28.1)
No recurrence	0 (0)	1 (100)	17 (42.5)	23 (71.9)

## Discussion

Previous studies have shown that combining clinical and genomic information allows for a more precise prognostic assessment than using either type of information alone in patients with breast cancer [12, 13]. This study focused on early recurrence within 3 years after surgery and revealed that clinical risk classification, when combined with a MammaPrint risk score, provided a more accurate assessment of the likelihood of early recurrence in HR +/HER2- early breast cancer patients. The study findings suggested that patients with both high clinical and genomic risk were at the highest risk for early recurrence, whereas those with both low clinical and genomic risk were at the lowest risk. Interestingly, patients with low clinical risk but high genomic risk appear to have an intermediate risk for early recurrence.

MammaPrint identifies genes related to distant metastasis through comprehensive genomic analysis rather than selecting genes known to be associated with prognosis [14, 15]. The genes in the MammaPrint assay are involved in early metastatic invasion processes, which consist of seven steps: growth and proliferation, angiogenesis, local invasion, intravasation, survival in circulation, extravasation, and microenvironmental adaptation at the metastatic site [16]. MammaPrint results had the greatest prognostic value for identifying patients at high risk for recurrence within 5 years after surgery in a validation study [16]. These findings aligned with the results of the current study, in which high-risk MammaPrint status was more frequently observed in patients with early recurrence.

In addition, a significantly greater percentage of patients in the early recurrence group than in the no recurrence group had Luminal B-type disease according to the Blueprint assay; basal-type disease was observed in only one patient in the no recurrence group, and the HER2-type disease was not observed in either group. In the MINDACT trial, prognostic outcomes varied by molecular subtype, with the Basal-type having the poorest prognosis, followed by the Luminal B, HER2, and Luminal A subtypes [5-year distant metastasis-free survival (DMFS): 90.4%, 92.7%, 94.3%, and 96.7%, respectively] [17]. In the NBRST trial, which aimed to demonstrate the utility of MammaPrint and BluePrint in the neoadjuvant setting, approximately 20% of the 426 cases pathologically classified as HR + HER2- were reclassified into non-luminal subtypes (85 cases as Basal-type and 2 cases as HER2-type) [18]. Furthermore, 80% of the cases classified as Basal-type experienced recurrence events within three years of diagnosis [18]. We hypothesized that the proportion of Basal-type cases would be the highest among patients with early recurrence. However, possibly due to the small sample size, Basal-type cases were rarely observed.

In this study, an association was observed between MammaPrint high risk results, Ki-67 levels, and nuclear grade. Previous reports have also shown a correlation between MammaPrint high risk results and a high Ki-67 index, as well as an association between a high Ki-67 index and the Nottingham histological grade [19]. On the other hand, when these clinicopathological factors were excluded, a MammaPrint high risk results was considered an independent prognostic marker.

In the present study, we did not evaluate whether clinical or genomic factors are predictive of systemic treatment outcomes. In the FLEX registry trial, patients with MammaPrint high-2–risk, BluePrint Luminal B, HR +/HER2- early breast cancer had a higher 3-year recurrence-free survival rate with anthracycline plus a taxane and cyclophosphamide than with a taxane and cyclophosphamide therapy in a nonrandomized cohort, and this tendency was not observed in patients with MammaPrint high-1–risk disease [20]. Although this study primarily evaluated prognostic factors, identifying predictive markers for the efficacy of individual therapies, including molecularly targeted agents, remains essential.

Although there are several commercially available gene expression profiling assays including Oncotype DX, we didn’t compare the performance of different assays in the present study. MammaPrint/BluePrint provides both prognostic risk stratification and molecular subtyping based on tumor biology, and we anticipate that the precise identification of tumors prone to early recurrence by MammaPrint/BluePrint may guide patient selection for intensified therapeutic strategies, including molecularly targeted agents.

Early recurrences were also observed among patients with both clinical low-risk and genomic low-risk disease, underscoring the challenge of identifying such cases and guiding appropriate treatment strategies. Further clinical studies are needed to optimize treatment, particularly for patients with clinical low-risk and genomic high-risk disease. More accurate identification of patients at elevated risk of early recurrence may enable better selection of candidates for adjuvant treatment intensification, such as CDK4/6 inhibitors. In addition, whether MammaPrint and BluePrint can serve not only as prognostic tools but also as predictive markers for the efficacy of specific therapies warrants evaluation in future prospective clinical trials. Such investigations will advance precision medicine and help refine therapeutic decision-making for patients with HR +/HER2 − breast cancer.

The primary limitations of this study are its retrospective nature and the small sample size. The availability of tissue specimens from the WJOG15721B trial was limited, and although 115 samples were submitted for MammaPrint and BluePrint testing, successful measurement was achieved in only 86 cases, resulting in an analysis success rate of approximately 75%. One possible reason for the low success rate is that the tissue samples used in this study were collected between 2012 and 2017, during which time the cold ischemia time and formalin fixation time were not as strictly controlled as they are today. As a result, the samples may not have been in an optimal condition for RNA analysis. Additionally, variations in the type and concentration of formalin used across different institutions and time points of sample collection may have influenced the results. Although a matched analysis was originally intended, only 86 samples were successfully measured, resulting in a number of unmatched cases and necessitating an unmatched analysis. This reduction in the number of evaluable samples represents sample attrition from the original study population and may have decreased the statistical power of the analysis. Furthermore, because the analysis was ultimately restricted to cases with successfully measured specimens, selection bias cannot be excluded, as these cases may not fully represent the overall study population. Therefore, the loss of the original matched design and the reduced sample size should be taken into account when interpreting the robustness and generalizability of our findings, and the results should be interpreted with appropriate caution. Despite these limitations, our study demonstrated meaningful results for current and future personalized medicine approaches.

## Conclusions

In this study, patients with early recurrence had a significantly greater prevalence of MammaPrint high-risk results and BluePrint Luminal B-type results than patients with no recurrence did. A potential association with the genetic risk score, molecular subtype, and early recurrence was suggested in patients with HR +/HER2- clinical stage II–III breast cancer.

## Supplementary Information

Below is the link to the electronic supplementary material.

Supplementary file1 (DOCX 48 KB)

## Data Availability

The datasets generated during and/or analyzed during the current study are available from the corresponding author upon reasonable request.

## References

[1] Ogiya A, Yamazaki K, Horii R, Shien T, Horimoto Y, Masuda N, et al. Post-relapse survival in patients with the early and late distant recurrence in estrogen receptor-positive HER2-negative breast cancer. Breast Cancer. 2017;24:473–82. 27628678 10.1007/s12282-016-0730-3

[2] Watanuki R, Sakai H, Takehara Y, Yoshida A, Hayashi N, Ozaki Y, et al. Risk factors for early recurrence in patients with hormone receptor-positive, HER2-negative breast cancer: a retrospective cohort study in Japan (WJOG15721B). Breast Cancer. 2025. 10.1007/s12282-025-01700-y. 40208504 10.1007/s12282-025-01700-y PMC12174269

[3] Cardoso F, van’t Veer LJ, Bogaerts J, Slaets L, Viale G, Delaloge S, et al. 70-gene signature as an aid to treatment decisions in early-stage breast cancer. N Engl J Med. 2016;375:717–29. 27557300 10.1056/NEJMoa1602253

[4] Piccart M, van ’t Veer LJ, Poncet C, Cardozo JMNL, Delaloge S, Pierga JY, et al. 70-gene signature as an aid for treatment decisions in early breast cancer: updated results of the phase 3 randomised MINDACT trial with an exploratory analysis by age. Lancet Oncol. 2021; 22: 476–488. 10.1016/S1470-2045(21)00007-3 33721561

[5] Krijgsman O, Roepman P, Zwart W, Carroll JS, Tian S, de Snoo FA, et al. A diagnostic gene profile for molecular subtyping of breast cancer associated with treatment response. Breast Cancer Res Treat. 2012;133:37–47. 21814749 10.1007/s10549-011-1683-z

[6] Mittempergher L, Delahaye LJMJ, Witteveen AT, Snel MHJ, Mee S, Chan BY, et al. Performance characteristics of the Blueprint® breast cancer diagnostic test. Transl Oncol. 2020;13:100756. 32208353 10.1016/j.tranon.2020.100756 PMC7097521

[7] Yamashita H, Ogiya A, Shien T, Horimoto Y, Masuda N, Inao T, et al. Clinicopathological factors predicting early and late distant recurrence in estrogen receptor-positive, HER2-negative breast cancer. Breast Cancer. 2016;23:830–43. 26467036 10.1007/s12282-015-0649-0

[8] Kennecke H, McArthur H, Olivotto IA, Speers C, Bajdik C, Chia SK, et al. Risk of early recurrence among postmenopausal women with estrogen receptor-positive early breast cancer treated with adjuvant tamoxifen. Cancer. 2008;112:1437–44. 18286526 10.1002/cncr.23320

[9] Cardoso F, Paluch-Shimon S, Senkus E, Curigliano G, Aapro MS, André F, et al. 5th ESO–ESMO international consensus guidelines for advanced breast cancer (ABC 5). Ann Oncol. 2020;31:1623–49. 32979513 10.1016/j.annonc.2020.09.010 PMC7510449

[10] Harbeck N, Rastogi P, Martin M, Tolaney SM, Shao ZM, Fasching PA, et al. Adjuvant abemaciclib combined with endocrine therapy for high-risk early breast cancer: updated efficacy and Ki-67 analysis from the monarchE study. Ann Oncol. 2021;32:1571–81. 34656740 10.1016/j.annonc.2021.09.015

[11] Johnston SRD, Harbeck N, Hegg R, Toi M, Martin M, Shao ZM, et al. Abemaciclib combined with endocrine therapy for the adjuvant treatment of HR+, HER2−, node-positive, high-risk, early breast cancer (monarchE). J Clin Oncol. 2020;38:3987–98. 32954927 10.1200/JCO.20.02514 PMC7768339

[12] Sparano JA, Gray RJ, Makower DF, Pritchard KI, Albain KS, Hayes DF, et al. Adjuvant chemotherapy guided by a 21-gene expression assay in breast cancer. N Engl J Med. 2018;379:111–21. 29860917 10.1056/NEJMoa1804710 PMC6172658

[13] Sparano JA, Gray RJ, Ravdin PM, Makower DF, Pritchard KI, Albain KS, et al. Clinical and genomic risk to guide the use of adjuvant therapy for breast cancer. N Engl J Med. 2019;380:2395–405. 31157962 10.1056/NEJMoa1904819 PMC6709671

[14] van ’t Veer LJ, Dai H, van de Vijver MJ, He YD, Hart AA, Mao M, et al. Gene expression profiling predicts clinical outcome of breast cancer. Nature. 2002; 415: 530–536. 10.1038/415530a 11823860

[15] van de Vijver MJ, He YD, van ’t Veer LJ, Dai H, Hart AA, Voskuil DW, et al. A gene-expression signature as a predictor of survival in breast cancer. N Engl J Med. 2002; 347: 1999–2009. 10.1056/NEJMoa021967 12490681

[16] Buyse M, Loi S, van ’t Veer L, Viale G, Delorenzi M, Glas AM, et al. Validation and clinical utility of a 70-gene prognostic signature for women with node-negative breast cancer. J Natl Cancer Inst. 2006;98:1183–1192. 10.1093/jnci/djj329 16954471

[17] Viale G, de Snoo FA, Slaets L, Bogaerts J, van ’t Veer L, Rutgers EJ, et al. Immunohistochemical versus molecular (BluePrint and MammaPrint) subtyping of breast carcinoma: outcome results from the EORTC 10041/BIG 3–04 MINDACT trial. Breast Cancer Res Treat. 2018; 167: 123–131. 10.1007/s10549-017-4509-9 28929359

[18] Whitworth P, Beitsch PD, Pellicane JV, Baron PL, Lee LA, Dul CL, et al. Age-independent preoperative chemosensitivity and 5-year outcome determined by combined 70- and 80-gene signature in a prospective trial in early-stage breast cancer. Ann Surg Oncol. 2022;29:4141–52. 35378634 10.1245/s10434-022-11666-2 PMC9174138

[19] Amezcua-Gálvez JE, Lopez-Garcia CA, Villarreal-Garza C, Lopez-Rivera V, Canavati-Marcos M, Santuario-Facio S, et al. Concordance between Ki-67 index in invasive breast cancer and molecular signatures: EndoPredict and MammaPrint. Mol Clin Oncol. 2022;17:132. 35949891 10.3892/mco.2022.2565 PMC9353786

[20] O’Shaughnessy J, Graham CL, Whitworth P, Beitsch PD, Osborne CRC, Rahman RL, et al. Association of MammaPrint index and 3-year outcome of patients with HR+HER2− early-stage breast cancer treated with chemotherapy with or without anthracycline. J Clin Oncol. 2024;42:511.

