# Supplementary material for: Characteristics of a multigene assay (MammaPrint/Blueprint) to predict early recurrence of hormone receptor-positive, HER2-negative breast cancer: a case‒control study (WJOG16722B)
Source: Breast Cancer. 2026 Mar 26;33(3):693–704. doi: 10.1007/s12282-026-01847-2 (PMC13124841; doi:10.1007/s12282-026-01847-2)
Supplement: Supplementary file 1 — Supplementary file1 (DOCX 48 KB) [file 12282_2026_1847_MOESM1_ESM.docx]

**Supplementary Table1.** Patient characteristics and clinicopathological features of the study population

|  | No recurrence  (n=42) | Early recurrence  (n=43) | Total  (n=85) |
| --- | --- | --- | --- |
| Comorbidity^*1^ | (n=31) | (n=28) |  |
| None | 22 (71.0) | 16 (57.1) | 38 |
| Malignant disease except breast cancer | 3 (9.7) | 1 (3.6) | 4 |
| Collage disease | 1 (3.2) | 0 (0) | 1 |
| Chronic liver disease | 0 (0) | 0 (0) | 0 |
| Diabetes | 0 (0) | 1 (3.6) | 1 |
| Hypertension | 1 (3.2) | 2 (7.1) | 3 |
| Renal dysfunction | 4 (12.9) | 2 (7.1) | 0 |
| Other | 6 (19.4) | 9 (32.1) | 15 |
| Diagnostic occasion |  |  |  |
| Symptom awareness | 25 (59.5) | 39 (90.7) | 64 |
| Detection by medical checkup | 7 (16.7) | 2 (4.7) | 9 |
| Accidental detection | 1 (2.3) | 0 (0) | 1 |
| Other | 9 (21.4) | 2 (4.7) | 11 |
| Bilateral breast cancer |  |  |  |
| No | 42 (100) | 42 (97.7) | 84 |
| Yes | 0 (0) | 1 (2.3) | 1 |
| Clinical T factor |  |  |  |
| T1 | 3 (7.1) | 4 (9.3) | 7 |
| T2 | 35 (83.3) | 35 (81.4) | 70 |
| T3 | 1 (2.3) | 2 (4.7) | 3 |
| T4 | 3 (7.1) | 2 (4.7) | 5 |
| Clinical N factor |  |  |  |
| N0 | 31 (73.8) | 27 (62.8) | 58 |
| N1 | 9 (21.4) | 14 (32.6) | 23 |
| N2 | 2 (4.8) | 2 (4.7) | 4 |
| N3 | 0 (0) | 0 (0) | 0 |
| Pathological T factor |  |  |  |
| T1 | 13 (31.0) | 8 (18.6) | 21 |
| T2 | 26 (61.9) | 26 (60.5) | 52 |
| T3 | 3 (7.1) | 6 (14.0) | 9 |
| T4 | 0 (0) | 1 (2.3) | 1 |
| Unknown | 0 (0) | 2 (4.7) | 2 |
| Pathological N factor |  |  |  |
| N0 | 16 (38.1) | 15 (34.9) | 31 |
| N1 | 19 (45.2) | 19 (44.2) | 38 |
| N2 | 4 (9.5) | 5 (11.6) | 9 |
| N3 | 3 (7.1) | 4 (9.3) | 7 |
| Pathological stage |  |  |  |
| I | 9 (21.4) | 3 (7.0) | 12 |
| IIA | 11 (26.2) | 14 (32.6) | 25 |
| IIB | 13 (31.0) | 13 (30.2) | 26 |
| IIIA | 6 (14.3) | 8 (18.6) | 14 |
| IIIB | 0 (0) | 1 (2.3) | 1 |
| IIIC | 3 (7.1) | 4 (9.3) | 7 |
| Histological type |  |  |  |
| Invasive cancer | 0 (0) | 1 (2.3) | 1 |
| Invasive ductal cancer | 39 (92.9) | 39 (90.7) | 78 |
| Special type | 3 (7.1) | 3 (7.0) | 6 |
| Invasive lobular carcinoma | 1 (2.3) | 2 (4.7) | 3 |
| Mucinous carcinoma | 2 (4.8) | 0 (0) | 2 |
| Invasive micropapillary carcinoma | 0 (0) | 1 (2.3) | 1 |
| Histological grade |  |  |  |
| 1 | 4 (9.5) | 1 (2.3) | 5 |
| 2 | 3 (7.1) | 3 (7.0) | 6 |
| 3 | 0 (0) | 2 (4.7) | 2 |
| Unknown | 35 (83.3) | 37 (86.0) | 72 |
| ER |  |  |  |
| <1% | 0 (0) | 0 (0) | 0 |
| 1-9% | 0 (0) | 0 (0) | 0 |
| ≥10% | 21 (50) | 25 (58.1) | 46 |
| Unknown | 21 (50) | 18 (41.9) | 39 |
| PgR |  |  |  |
| <1% | 3 (7.1) | 2 (4.7) | 5 |
| ≥1% | 10 (23.8) | 14 (32.6) | 24 |
| Unknown | 29 (69.0) | 27 (62.8) | 56 |
| HER2 |  |  |  |
| 0 | 20 (47.6) | 20 (46.5) | 40 |
| 1+ | 17 (40.5) | 17 (39.5) | 34 |
| 2+ | 5 (11.9) | 6 (14.0) | 11 |
| Ki-67 |  |  |  |
| <14% | 7 (16.7) | 3 (7.0) | 10 |
| 14-29% | 5 (11.9) | 4 (9.3) | 9 |
| ≥30% | 4 (9.5) | 15 (34.9) | 19 |
| Unknown | 26 (61.9) | 21 (48.8) | 47 |
| Lymphatic invasion |  |  |  |
| No | 21 (50) | 13 (30.2) | 34 |
| Yes | 21 (50) | 30 (69.8) | 51 |
| Vascular invasion |  |  |  |
| No | 36 (85.7) | 37 (86.0) | 73 |
| Yes | 5 (11.9) | 6 (14.0) | 11 |
| Unknown | 1 (16.7) | 0 (0) | 1 |
| Pathological therapeutic response ^*2^ |  |  |  |
| Grade0 | 0 (0) | 2 (16.7) | 2 |
| Grade1 | 5 (83.3) | 6 (50) | 11 |
| Grade2 | 1 (16.7) | 4 (33.3) | 5 |
| Grade3 | 0 (0) | 0 (0) | 0 |

*1 Data represent aggregated results from 59 cases obtained from three institutions.

*1 Data were tabulated for the patients who received preoperative chemotherapy or preoperative endocrine therapy.

**Supplementary Table2.** Perioperative treatment details of the study population

|  | No recurrence  (n=42) | Early recurrence  (n=43) | Total  (n=85) |
| --- | --- | --- | --- |
| Neoadjuvant endocrine therapy |  |  |  |
| No | 40 (95.2) | 42 (97.7) | 82 |
| Yes | 2 (4.8) | 1 (2.3) | 3 |
| AI | 2 (4.8) | 1 (2.3) | 3 |
| Adjuvant endocrine therapy |  |  |  |
| No | 0 (0) | 0 (0) | 0 |
| Yes | 42 (100) | 43 (100) | 85 |
| SERM alone | 15 (35.7) | 22 (51.2) | 37 |
| AI alone | 20 (47.6) | 14 (32.6) | 34 |
| SERM+LHRH agonist | 5 (11.9) | 5 (11.6) | 10 |
| SERM → AI | 1 (2.4) | 0 (0) | 1 |
| Others | 1 (2.4) | 2 (4.7) | 3 |

Abbreviations: SERM, selective estrogen receptor modulator; LHRH agonist, luteinizing hormone-releasing hormone agonist; AI, aromatase inhibitor

**Supplementary table 3.** Patient characteristics and clinicopathological features in assay-successful and assay-unsuccessful cases

|  | Unsuccessful analysis  (n=29) | Successful analysis  (n=85) | P value^*1^ |
| --- | --- | --- | --- |
| Age, years |  |  |  |
| Median (range) | 49 (35-73) | 48 (32-86) | 0.7996 |
| Performance status^*2^ | (n=11) | (n=50) |  |
| 0 | 11 (100) | 50（100） | - |
| 1 | 0（0） | 0（0） |  |
| Comorbidity^*3^ | (n=11) | (n=59) |  |
| None | 6 (54.5) | 38 (64.4) | 0.5343 |
| Malignant disease except breast cancer | 0 (0) | 4 (6.8) | 0.3738 |
| Collage disease | 0 (0) | 1 (1.7) | 0.6636 |
| Chronic liver disease | 0 (0) | 0 (0) | - |
| Diabetes | 0 (0) | 1 (1.7) | 0.6636 |
| Hypertension | 2 (18.2) | 3 (5.1) | 0.1215 |
| Renal dysfunction | 0 (0) | 0 (0) | - |
| Other | 3 (27.3) | 15 (25.4) | 0.8975 |
| Menopausal status |  |  |  |
| Premenopausal | 17 (58.6) | 48 (56.5) | 0.8338 |
| Postmenopausal | 12 (41.4) | 36 (42.4) |  |
| Unknown | 0 (0) | 1 (1.2) |  |
| Diagnostic occasion |  |  |  |
| Symptom awareness | 24 (82.8) | 64 (75.3) | 0.7551 |
| Detection by medical checkup | 3 (10.3) | 9 (10.6) |  |
| Accidental detection | 0 (0) | 1 (1.21) |  |
| Other | 2 (6.9) | 11 (12.9) |  |
| Bilateral breast cancer |  |  |  |
| No | 27 (93.1) | 84 (98.8) | 0.0966 |
| Yes | 2 (6.9) | 1 (1.2) |  |
| Clinical T factor |  |  |  |
| T1 | 2 (6.9) | 7 (8.2) | 0.0442 |
| T2 | 18 (62.1) | 70 (82.4) |  |
| T3 | 4 (13.8) | 3 (3.5) |  |
| T4 | 5 (17.2) | 5 (5.9) |  |
| Clinical N factor |  |  |  |
| N0 | 11 (37.9) | 58 (68.2) | 0.0002 |
| N1 | 10 (34.5) | 23 (27.1) |  |
| N2 | 3 (10.3) | 4 (4.7) |  |
| N3 | 5 (17.2) | 0 (0) |  |
| Clinical stage |  |  |  |
| IIA | 9 (31.0) | 63 (74.1) | <0.0001 |
| IIB | 6 (20.7) | 11 (12.9) |  |
| IIIA | 5 (17.2) | 6 (7.1) |  |
| IIIB | 4 (13.8) | 5 (5.9) |  |
| IIIC | 5 (17.2) | 0 (0) |  |
| Pathological T factor |  |  |  |
| T0 | 1 (3.4) | 0 (0） | 0.0006 |
| T1 | 3 (10.3） | 21 (24.7) |  |
| T2 | 12 (41.4） | 52 (61.2) |  |
| T3 | 6 (20.7) | 9 (10.6) |  |
| T4 | 0 (0) | 1 (1.2) |  |
| Unknown | 7 (24.1) | 2 (2.4) |  |
| Pathological N factor |  |  |  |
| N0 | 9 (31.0) | 31 (36.5) | 0.0266 |
| N1 | 8 (27.6) | 38 (44.7) |  |
| N2 | 10 (34.5) | 9 (10.6) |  |
| N3 | 2 (6.9) | 7 (8.2) |  |
| Pathological stage |  |  |  |
| I | 1 (3.4) | 12 (14.29） | 0.0502 |
| IIA | 8 (27.6） | 25 (29.4） |  |
| IIB | 4 (13.8） | 26 (30.6） |  |
| IIIA | 12 (41.4） | 14 (16.5） |  |
| IIIB | 0 (0) | 1 (1.2） |  |
| IIIC | 2 (6.9) | 7 (8.2） |  |
| Unknown | 2 (6.9) | 0 (0) |  |
| Histological type |  |  |  |
| Invasive cancer | 0 (0） | 1 (1.2） | 0.0890 |
| Invasive ductal cancer | 22 (75.9） | 78 (91.8） |  |
| Special type | 6 (20.7） | 6 (7.1） |  |
| Invasive lobular carcinoma | 5 (17.2) | 3 (3.5) |  |
| Mucinous carcinoma | 0 (0) | 2 (2.4) |  |
| Invasive micropapillary carcinoma | 1 (3.4) | 1(1.2) |  |
| Unknown | 1 (3.4) | 0 (0) |  |
| Nuclear grade |  |  |  |
| 1 | 11 (37.9) | 26 (30.6) | 0.0031 |
| 2 | 8 (27.6) | 37 (43.5) |  |
| 3 | 3 (10.3) | 19 (22.4) |  |
| Unknown | 7 (24.1) | 3 (3.5) |  |
| Histological grade |  |  |  |
| 1 | 1 (3.4) | 5 (5.9） | 0.5330 |
| 2 | 3 (10.3） | 6 (7.1） |  |
| 3 | 2 (6.9） | 2 (2.4） |  |
| Unknown | 23 (79.3) | 72 (84.7) |  |
| ER |  |  |  |
| <1% | 0 (0） | 0 (0） | 0.0023 |
| 1-9% | 2 (6.9） | 0 (0) |  |
| ≥10% | 22 (75.9） | 46 (54.1） |  |
| Unknown | 5 (17.2） | 39 (45.9） |  |
| PgR |  |  |  |
| <1% | 1 (3.4) | 5 (5.9) | 0.8597 |
| ≥1% | 9 (31.0） | 24 (28.2） |  |
| Unknown | 19 (65.5） | 56 (65.9） |  |
| HER2 |  |  |  |
| 0 | 17 (58.6） | 40 (47.1） | 0.3049 |
| 1+ | 7 (24.1） | 34 (40.0） |  |
| 2+ | 5 (17.2） | 11 (12.9） |  |
| Ki-67 |  |  |  |
| <14% | 2 (6.9） | 10 (11.8） | 0.2080 |
| 14-29% | 4 (13.8） | 9 (10.6） |  |
| ≥30% | 2 (6.9） | 19 (22.4） |  |
| Unknown | 21 (72.4） | 47 (55.3） |  |
| Lymphatic invasion |  |  |  |
| No | 11 (37.9） | 34 (40.0） | 0.9466 |
| Yes | 17 (58.6) | 51 (60.0） |  |
| Unknown | 1 (3.4) | 0 (0) |  |
| Vascular invasion |  |  |  |
| No | 26 (89.7) | 73 (85.9) | 0.3944 |
| Yes | 2 (6.9) | 11 (12.9) |  |
| Unknown | 1 (3.4) | 1 (1.2) |  |
| Pathological tumor size (cm) |  |  |  |
| <2 | 4 (13.8) | 16 (18.8) | 0.2035 |
| 2 to less than 5 | 12 (41.4) | 57 (67.1) |  |
| ≥5 | 6 (20.7) | 10 (11.8) |  |
| Unknown | 7 (24.1) | 2 (2.4) |  |
| Number of pathological lymph node metastases |  |  |  |
| 0 | 9 (31.0） | 31 (36.5） | 0.0266 |
| 1-3 | 8 (27.6） | 38 (44.7） |  |
| 4-9 | 10 (34.5） | 9 (10.6） |  |
| ≥10 | 2 (6.9） | 7 (8.2） |  |
| Pathological therapeutic response ^*4^ |  |  |  |
| Grade0 | 1 (5.6) | 2 (11.1） | 0.3949 |
| Grade1 | 14 (77.8） | 11 (61.1） |  |
| Grade2 | 2 (11.1) | 5 (27.8） |  |
| Grade3 | 1 (5.6） | 0 (0) |  |

*1 Categorical variables were analyzed using the chi-square test, and continuous variables were analyzed using the Wilcoxon rank-sum test.

*2 Data represent aggregated results from 61 cases obtained from two institutions.

*3 Data represent aggregated results from 70 cases obtained from three institutions.

*2 Data were tabulated for the patients who received preoperative chemotherapy or preoperative endocrine therapy.
